# Risk Factors for First-Attempt Suicide Fatalities: Evidence From the MCOSUL Cohort

**DOI:** 10.62641/aep.v54i3.2220

**Published:** 2026-06-15

**Authors:** Eugènia Nicolau-Subires, María Irigoyen-Otiñano, Teresa Jové-Miró, Laura Arenas-Pijoan, Rubèn Gavilà-Esquerdo, Samuel Pámpols-Pérez, Anna Gallart-López, Ana Marina Lao-García, Núria Domènech-Martínez, Andrea Jiménez-Mayoral, Vicent Llorca-Bofí

**Affiliations:** ^1^Group of Biological Functionings of Mental Disorders, Institute for Biomedical Research in Lleida (IRB), 25198 Lleida, Spain; ^2^Department of Psychiatry Hospital Universitario Santa María Lérida, 25198 Lleida, Spain; ^3^Centro de Investigación Biomédica en Red de Salud Mental (CIBERSAM), 28029 Madrid, Spain; ^4^Universitat de Lleida, 25198 Lleida, Spain; ^5^Department of Psychiatry, Hospital Clinic de Barcelona, 08006 Barcelona, Spain

**Keywords:** suicide, Suicide, Attempted, cohort studies, risk factors, mental disorders

## Abstract

**Background:**

Individuals who die by suicide may differ depending on whether the fatal act is their first attempt or follows previous non-fatal attempts. Understanding these differences may help refine prevention strategies.

**Methods:**

We conducted a cross-sectional analysis within the MCOSUL Cohort, a clinical registry of all patients treated for a suicide attempt in two general hospitals in Lleida, Spain. We included all cohort members who died by suicide between January 2009 and December 2022. Sociodemographic, clinical, and method-related variables were compared between first-attempt and repeat-attempt fatalities. Variables with *p* < 0.20 in bivariate analyses were entered into a multivariable logistic regression model to identify independent predictors of first-attempt fatalities.

**Results:**

Among 117 suicide deaths, 38 (32.5%) occurred at the first attempt and 79 (67.5%) after one or more prior non-fatal attempts. Repeat-attempt fatalities had a mean of 1.89 (SD = 1.73) attempts from cohort entry to death. In multivariable analysis, unemployment (OR = 0.17, 95% CI: 0.04–0.66, *p* = 0.011) and recognized disability (OR = 0.20, 95% CI: 0.04–0.80, *p* = 0.035) were less frequent among first-attempt fatalities, indicating they were more common in those who died after repeated attempts. No significant differences were found for age, sex, alcohol use, psychiatric diagnosis, or fatal attempt method.

**Conclusions:**

Unemployment and recognized disability (formal legal/administrative disability certification issued by relevant regional authorities, which may encompass physical and/or psychiatric disabilities) appear to characterize individuals who die by suicide after a series of non-fatal attempts, suggesting a subgroup with a more prolonged suicidal course that may benefit from sustained engagement and prevention strategies.

## Introduction

Suicide is a crucial public health problem with a serious impact on individuals, families, and societies. Suicide attempts have been recognized as a significant risk factor for both repeat and completed suicides [1]. Suicide attempts are more common than completed suicides, and there is some evidence that suicide attempts and completions are two distinct subgroups [2]. A study has reported that 92.3% of suicides were completed during the first or second attempts [3]. Approximately 77% of individuals who died by suicide had contact with a primary care physician in the previous year, and about 45% in the month prior [4]. However, no comparative data have been published on the pre-monitoring of those who attempt and complete suicide in primary care or mental health services. This means that preventive strategies focused on individuals with a high frequency of suicide attempts may not be sufficient to prevent suicide completion [5].

Previous research has primarily examined risk factors for suicide attempts and completed suicide as separate or aggregated outcomes [6, 7]. However, relatively little attention has been paid to differences between individuals who die on their first suicide attempt and those who die after one or more prior non-fatal attempts. This distinction is clinically and theoretically relevant, as it may reflect different trajectories of suicidal behavior, patterns of healthcare contact, and windows of opportunity for intervention [8].

In addition, most available evidence comes from cross-sectional or retrospective designs without longitudinal follow-up, limiting the ability to characterize the progression from index attempt to death. Therefore, examining these subgroups within a longitudinal clinical cohort may provide a more nuanced understanding of suicide pathways [6].

This study aims to describe the sociodemographic and clinical characteristics of patients who die by suicide, distinguishing between those who die on their first recorded suicide attempt within the cohort and those who die after several previous attempts. By explicitly comparing these subgroups within a longitudinal framework, this study extends prior literature and seeks to inform more tailored prevention strategies.

## Methods

### Study Design and Participants

This study has an ambispective cohort design, combining retrospective and prospective data within the MCOSUL cohort. This cohort consists of individuals from the population served by Hospital Universitari Arnau de Vilanova and Santa Maria University Hospitals in Lleida, Catalonia, Spain. The hospitals share the Department of Psychiatry and are the exclusive providers of round-the-clock psychiatric care in the entire province of Lleida, offering universal public health coverage to all residents of the region, including children, adolescents, and adults. The hospital serves an area of influence encompassing 447,343 people as of 2023 [9].

Retrospective data were obtained from electronic health records (EHR) dating back to 2009, including information on the index episode and subsequent events when available. Prospective follow-up was conducted systematically after cohort establishment through continuous monitoring of patients using EHR.

The integration of retrospective and prospective components was ensured through a unified electronic health record system covering the entire catchment area. Follow-up completeness was maximized because the participating hospitals are the exclusive providers of psychiatric emergency care in the region. In addition, trained psychiatrists conducted regular standardized reviews of clinical records to ensure data quality and consistency.

Participants are recruited following a suicide attempt and are monitored over time.

The cohort systematically collects sociodemographic, clinical, and follow-up information starting from the index episode, defined as the first suicide attempt treated by the Department of Psychiatry and meeting Silverman’s criteria [10]. These criteria specify a suicide attempt as a self-inflicted, potentially harmful behavior with a non-fatal outcome, where there is evidence (explicit or implicit) of intent to die. Patients are subsequently followed until the present date or death, and all subsequent suicide attempts and relevant outcomes are recorded.

Data following the index episode are collected by trained psychiatrists through regular reviews of the patients EHR within the cohort. Several key patient outcomes after discharge are monitored. First, the date of the initial outpatient follow-up visit is noted. Second, we examine whether the patient experiences any suicidal reattempts during the follow-up period. If such reattempts occur, the date and details of the attempts, such as the method used and severity, are documented. Finally, if death occurs, the date and cause of death are recorded.

### Ethical Considerations

The study was conducted in accordance with the ethical principles of the Declaration of Helsinki [11]. Approval was obtained from the Ethics and Clinical Research Committee of Hospital Universitari Arnau de Vilanova (CEIC-1540). The ethics committee covers the study from the existence of the center’s digital clinical records dating back to 2009 and in accordance with the ambispective nature of the study. That informed consent was waived by the ethics committee due to the retrospective use of anonymized data.

### Variables

Variables were extracted from the MCOSUL database and medical records. Sociodemographic data included sex (male/female), age at death (years), marital status (single, married/coupled, divorced, widowed), and occupational status (employed, unemployed, inactive due to disability or retirement). Clinical variables included recognized disability (yes/no), degree of disability (percentage), family history of suicide (yes/no), and psychiatric diagnoses according to DSM-IV criteria, grouped into affective disorders, anxiety disorders, personality disorders, psychotic disorders, substance use disorders, and other disorders. Substance use variables included alcohol use, cannabis use, and polysubstance use—defined as the regular use of three or more psychoactive substances other than tobacco and methadone [12].

The method of the fatal suicide attempt was classified into individual categories (e.g., hanging, defenestration, firearms, self-immolation, drowning, self-poisoning) and into grouped categories: violent methods (e.g., hanging, defenestration, firearms, self-immolation, drowning, severe cutting, and other violent mechanisms), non-violent methods (self-poisoning with sedatives or non-sedatives), and unknown.

### Outcomes

The primary outcome was the condition of death:

• First-attempt fatalities—death occurred during the first suicide attempt recorded in the MCOSUL Cohort.

• Repeat-attempt fatalities—death occurred after one or more non-fatal attempts recorded in the cohort.

This definition reflects the availability of data within the registry and may not capture suicide attempts occurring prior to cohort entry or outside the healthcare system.

### Statistical Analysis

Continuous variables were summarized using means and standard deviations (SD) as appropriate. Categorical variables were expressed as counts and percentages. Comparisons between first-attempt and repeat-attempt fatalities were performed using the Student’s t-test or Wilcoxon rank-sum test for continuous variables and the Chi-square test or Fisher’s exact test for categorical variables.

A multivariable logistic regression model was used to identify factors independently associated with first-attempt suicide fatalities. Variables with *p* < 0.20 in bivariate analyses were considered for inclusion, together with variables deemed clinically relevant (including age and sex), in order to balance statistical and conceptual considerations.

Given the limited number of events, the number of variables included in the final model was restricted to reduce the risk of overfitting. Model assumptions were assessed, including collinearity diagnostics and overall goodness of fit.

Odds ratios (OR) with 95% confidence intervals (CI) were reported.

## Results

### Characteristics of the Study Population

A total of 117 suicide fatalities were included in the analysis. The mean age at death was 48 years (SD = 18), and 41% were female. The most common occupational status was unemployment (44.4%), and 18.8% of cases had a recognized disability, with a mean disability rating of 50% (SD = 17). Regarding psychiatric history, the most frequent primary diagnoses were affective disorders (47.9%) and substance use disorders (23.9%). Alcohol use was reported in 31.6% of cases, cannabis use in 3.4%, and polysubstance use in 11.1% (Table 1).

**Table 1.** **Sociodemographic and clinical characteristics of first-attempt and repeat-attempt suicide fatalities**.

Characteristics		Total (N = 117)	First-attempt fatalities	Repeat-attempt fatalities	*p*-value
			(n = 38; 32.5%)	(n = 79; 67.5%)	
Age (years), mean (SD)		48 (18)	51 (19)	46 (17)	0.210
Female, n (%)		48 (41.0)	11 (28.9)	37 (46.8)	0.074
Marital status, n (%)					0.913
	Single	41 (35.0)	14 (36.8)	27 (34.2)	
	Married or coupled	41 (35.0)	14 (36.8)	27 (34.2)	
	Divorced	27 (23.1)	8 (21.1)	19 (24.1)	
	Widower	8 (6.8)	2 (5.3)	6 (7.6)	
Occupation, n (%)					0.008*
	Employed	17 (14.5)	8 (21.1)	9 (11.4)	
	Unemployed	52 (44.4)	9 (23.7)	43 (54.4)	
	Inactive (disability or retired)	48 (41.0)	21 (55.3)	27 (34.2)	
	Recognized disability, n (%)	22 (18.8)	3 (7.9)	19 (24.1)	0.041*
	Recognized disability rating, mean (SD)	50 (17)	48 (16)	50 (17)	0.875
	Family history of death by suicide, n (%)	7 (6.0)	2 (5.3)	5 (6.3)	0.922
Psychiatric diagnosis, n (%)					0.817
	Affective disorders	56 (47.9)	18 (47.4)	38 (48.1)	
	Anxiety disorders	9 (7.7)	2 (5.3)	7 (8.9)	
	Personality disorders	10 (8.5)	4 (10.5)	6 (7.6)	
	Psychotic disorders	6 (5.1)	1 (2.6)	5 (6.3)	
	Substance use disorders	28 (23.9)	10 (26.3)	18 (22.8)	
	Other	8 (6.8)	3 (7.9)	5 (6.3)	
Alcohol use, n (%)		37 (31.6)	8 (21.1)	29 (36.7)	0.106
Cannabis use, n (%)		4 (3.4)	0 (0.0)	4 (5.1)	0.314
Polysubstance use, n (%)		13 (11.1)	4 (10.5)	9 (11.4)	0.989

Significant results (*p* < 0.05) are marked with an asterisk.

### Comparison Between First-Attempt and Repeat-Attempt Fatalities

Of the total cases, 38 deaths (32.5%) occurred during the first suicide attempt and 79 (67.5%) after a repeat attempt. Among repeat-attempt fatalities, the mean number of attempts from the index episode of the MCOSUL cohort until death was 1.89 (SD = 1.73).

Categorical variables were compared using chi-square tests. When an overall significant association was detected, pairwise comparisons between groups were performed using two-proportion z-tests. To control for multiple comparisons, a Bonferroni correction was applied, and adjusted p-values were reported.

There was an overall difference in occupation distribution between groups (*p* = 0.008). Pairwise comparisons showed that unemployment was more frequent among repeat-attempt fatalities (54.4% vs. 23.7%, *p* = 0.0017), whereas differences in employed and inactivity status were not statistically significant after correction. First-attempt fatalities were also less frequent among individuals with a recognized disability (7.9% vs. 24.1%, *p* = 0.041). No significant group differences were observed for age, sex, marital status, psychiatric diagnosis, alcohol use, or other toxic substance use (Table 1).

Violent methods accounted for 60% of all fatalities, followed by non-violent methods (16%) and unknown methods (24%). The most frequent individual methods were hanging (25%), defenestration (20%), and pharmaceutical poisoning with sedatives (15%). Other methods included drowning (6.8%), firearms (4.3%), self-immolation (2.6%), and cutting (0.9%). Pharmaceuticals other than sedatives accounted for 1.7% of cases, and other unspecified methods for 0.9%. No significant differences were observed in the distribution of methods between first-attempt and repeat-attempt fatalities.

### Predictors of First-Attempt Suicide Fatalities

In the multivariable logistic regression model, being unemployed (OR = 0.17, 95% CI: 0.04–0.66, *p* = 0.011) and having a recognized disability (OR = 0.20, 95% CI: 0.04–0.80, *p* = 0.035) were independently associated with lower odds of dying on the first suicide attempt, compared with employed individuals and those without recognized disability, respectively. Age, sex, alcohol use, and being inactive due to disability or retirement (without formal recognition) were not significantly associated with the outcome (Table 2).

**Table 2.** **Logistic regression analysis of factors associated with first-attempt suicide fatalities**.

Term	*p*-value	OR (95% CI)
Age (years)	0.4196	1.01 (0.99–1.04)
Female	0.094	0.46 (0.18–1.13)
Unemployed	0.011*	0.17 (0.04–0.66)
Inactive (disability or retired)	0.376	0.56 (0.15–2.02)
Alcohol use	0.715	1.23 (0.40–3.74)
Recognized disability	0.035*	0.20 (0.04–0.80)

OR, odds ratio; CI, confidence interval; *p*-values from Wald test; N = 117. Reference category: male gender, employed, no alcohol use, no recognized disability. Significant results (*p* < 0.05) are marked with an asterisk.

## Discussion

This study investigated factors associated with dying by suicide at the first attempt versus after one or more prior non-fatal attempts in the MCOSUL Cohort, a clinical registry of individuals treated for suicide behaviour. Attempting to identify differences between patients who die on the first attempt or after several attempts may be an opportunity to establish more specific preventive measures in these high-risk populations [13, 14, 15, 16].

In our sample, unemployment and recognized disability were more common among individuals who died after repeated non-fatal attempts [17, 18]. Although this pattern may suggest a subgroup with a more prolonged or complex suicidal trajectory, this interpretation should be made cautiously. Alternative explanations include differences in healthcare utilization, greater likelihood of documentation of prior attempts, and survival bias [19, 20]. Therefore, these variables should be interpreted as correlates rather than causal markers.

A key methodological issue concerns the definition of first-attempt fatalities. In this study, this category refers to the first attempt recorded within the MCOSUL cohort, rather than the first lifetime suicide attempt. Consequently, some individuals may have been misclassified if previous attempts occurred outside the healthcare system or prior to cohort entry. This potential misclassification bias may have attenuated differences between groups and should be considered when interpreting the findings.

We did not observe significant differences between groups in terms of age, sex, psychiatric diagnosis, substance use, or suicide method. However, given the relatively small sample size—particularly the number of first-attempt fatalities (n = 38)—the study may have been underpowered to detect meaningful differences. These results should not be interpreted as evidence of absence of association.

The limited number of outcome events also constrains the multivariable analysis. Although we restricted the number of predictors and included clinically relevant variables, the risk of overfitting cannot be excluded. Therefore, the model results should be interpreted with caution.

Another important limitation is the absence of key clinical and psychosocial variables, including treatment history, medication adherence, access to mental health services, and social support. These factors are likely to influence both suicidal behavior and the probability of repeated attempts, and their absence may result in residual confounding.

Despite these limitations, the study has several strengths, including the use of a real-world clinical cohort with longitudinal follow-up and systematic data collection. The ambispective design enables reconstruction of patient trajectories over time, which is rarely available in suicide research

## Conclusions

Unemployment and recognized disability were more frequent among individuals who died by suicide after repeated non-fatal attempts. These factors may reflect more complex clinical trajectories or differences in healthcare engagement, although alternative explanations such as recording bias and survival effects should be considered. Differentiating between first-attempt and repeat-attempt fatalities may contribute to a more nuanced understanding of suicide risk and inform targeted prevention strategies.

## Availability of Data and Materials

The data that support the findings of this study are not publicly available due to ethical and legal restrictions related to the protection of personal and sensitive data. Anonymized data are available from the corresponding author upon reasonable request for research purposes.
